# A comparison of three interactive examination designs in active learning classrooms for nursing students

**DOI:** 10.1186/s12912-021-00575-6

**Published:** 2021-04-09

**Authors:** Linda Ahlstrom, Christopher Holmberg

**Affiliations:** 1 Institute of Health and Care Sciences, Section of Learning and Leadership for Health Care Professionals, University of Gothenburg, Arvid Wallgrens Backe, Box 457, 405 30 Gothenburg, Sweden; 2 Department of Orthopedics, Sahlgrenska University Hospital, Gothenburg, Sweden; 3 Department of Psychotic Disorders, Sahlgrenska University Hospital, Gothenburg, Sweden

**Keywords:** Active learning, Active learning classroom, Digital education, Formative assessment, Interactive examination, Nursing education, Quality improvement

## Abstract

**Background:**

Despite the advantages of using active learning strategies in nursing education, researchers have rarely investigated how such pedagogic approaches can be used to assess students or how interactive examinations can be modified depending on circumstances of practice (e.g., in online education).

**Aims:**

The aim was to compare three interactive examination designs, all based on active learning pedagogy, in terms of nursing students’ engagement and preparedness, their learning achievement, and instructional aspects.

**Methods:**

A comparative research design was used including final-year undergraduate nursing students. All students were enrolled in a quality improvement course at a metropolitan university in Sweden. In this comparative study to evaluate three course layouts, participants (Cohort 1, *n =* 89; Cohort 2, *n =* 97; Cohort 3, *n =* 60) completed different examinations assessing the same course content and learning objectives, after which they evaluated the examinations on a questionnaire in numerical and free-text responses. Chi-squared tests were conducted to compare background variables between the cohorts and Kruskal–Wallis H tests to assess numerical differences in experiences between cohorts. Following the guidelines of the Good Reporting of a Mixed Methods Study (GRAMMS), a sequential mixed-methods analysis was performed on the quantitative findings, and the qualitative findings were used complementary to support the interpretation of the quantitative results.

**Results:**

The 246 students who completed the questionnaire generally appreciated the interactive examination in active learning classrooms. Among significant differences in the results, Cohort 2 (e.g., conducted the examination on campus) scored highest for overall positive experience and engagement, whereas Cohort 3 (e.g., conducted the examination online) scored the lowest. Students in Cohort 3 generally commended the online examination’s chat function available for use during the examination.

**Conclusions:**

Interactive examinations for nursing students succeed when they are campus-based, focus on student preparation, and provide the necessary time to be completed.

**Supplementary Information:**

The online version contains supplementary material available at 10.1186/s12912-021-00575-6.

## Background

Active learning classrooms (ALC) employ various designs and approaches to support teaching and learning in an atmosphere conducive to actively engaging students in their own learning [1]. Attributes of active learning include cooperative learning, which requires students’ participation; problem-based learning, which promotes student engagement; and interactive learning [1]. In all active learning approaches, educators act as facilitators of knowledge acquisition, not one-way providers of knowledge [2, 3]. A systematic review has shown that active learning strategies provide student-centered approaches to learning and excel in engaging nursing students in understanding the complexities of contemporary health care [4]. In support, Matsuda et al. [5] observed that when students actively engage and connect with course content, their overall learning achievement improves. In other work, active learning has not only improved students’ perceptions of self-efficacy and inclusiveness in the classroom [6] but also promoted their ability to think critically and perform better on examinations [7, 8]. Beyond that, in a two-year study involving more than 35,000 units of data about students’ perceptions, students thought that courses using active learning as the principal learning strategy encouraged their creativity and innovation more than courses using traditional designs [9].

Despite research showing that nurse educators have generally adopted active learning strategies, the ways in which active learning can be used in examinations remain unclear [10]. Such knowledge matters, however, especially in view of research suggesting that formative assessments, wherein educators performed in-process evaluations of students’ knowledge, allow examining more complex learning objectives than summative examinations [10, 11]. For example, assessing complex learning objectives in subjects such as quality improvement (QI) ideally involves assessing students’ products and their processes [12, 13]. Added to that, interactive and formative examinations that consider students’ processes and progress are valuable because they target higher-order skills, including meaningful analysis [11, 14]. Among their practical advantages, interactive and formative assessments are efficient as they have a combined focus on learning and examination. This is important, as research indicate that there is a general concern for content saturation in nursing education [15].

Having access to digital means of implementing active learning strategies and interactive examinations is pivotal in distance education and when managing unforeseen events (e.g., the COVID-19 pandemic), both of which demand educators to keep abreast of evolving technologies [16, 17]. In addition, because telemedicine and digitalization play increasingly major roles in delivering high-quality health care, developing the best nursing practices requires nursing students to be familiar with using such interactions to communicate [18]. Such familiarity entails not only experience with the technology but also considering potential changes in group dynamics and interactions when communicating via digital technologies instead of face-to-face communication. On top of that, students’ sense of involvement and active engagement are key factors to their success in online education [16]. Even so, the literature reveals a lack of evidence about student nurses’ experiences with online education using digital technologies [16].

Against that background, the aim of this study was to compare three interactive examination designs, all based on active learning pedagogy, in terms of nursing students’ engagement and preparedness, their learning achievement, and instructional aspects.

## Methods

### Design

For this study, we adopted a sequential, mixed-methods, comparative research design [19, 20] deemed relevant to our aim of comparing students’ experiences with exposure to similar course content but with different active learning designs. We followed the guidelines of the Good Reporting of a Mixed Methods Study (GRAMMS) in outlining how the quantitative and qualitative components would be integrated and how their unique contributions relate to each other [21], as detailed in Supplementary File 1.

**Fig. 1:**
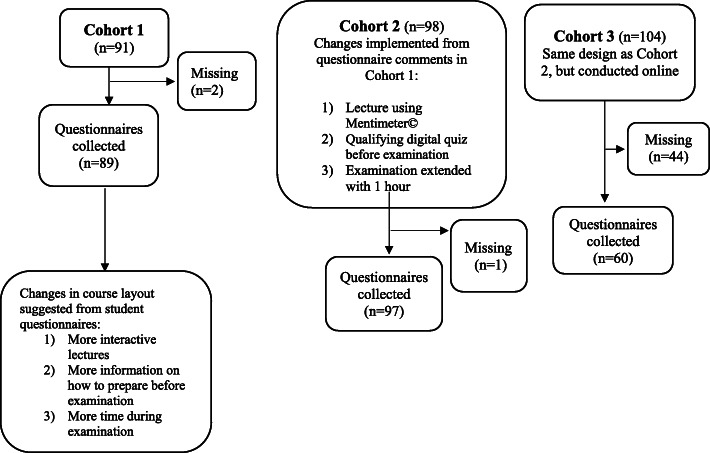
Illustration of the three student cohorts. Missing refers to students attending the examination but did not complete the questionnaires or submitted blank questionnaires

We evaluated an active learning course layout (Cohort 1, Fall Semester 2019) with a questionnaire completed students, the quantitative and qualitative results of which we consulted in modifying the course layout that we also evaluated (Cohort 2, Spring Semester 2020) with the same questionnaire. Due to the transition to online teaching required by the COVID-19 pandemic, we also evaluated a course layout that was implemented entirely online (Cohort 3, Fall Semester 2020), as shown in Fig. 1.

### Setting

We conducted our study with three cohorts of final-year undergraduate nursing students in a QI course at a metropolitan university in Sweden.

### Quality improvement course

We performed the interactive examination in a QI course offered during the last semester of a three-year undergraduate program. Given the dynamic nature of QI classes, interactive assessment methods are ideal for courses in that subject area [22], where learning achievements tend to include individual aspects (e.g., problem-solving abilities and critical thinking skills), interpersonal aspects (e.g., group work), and aspects of project management, including designing and managing projects in health care settings and using relevant theories and tools [23]. Because QI is a core competency of nursing and an important part of nursing curriculum [13, 24], nursing students need knowledge of QI methods and tools to apply clear goal-setting strategies, improve processes, implement suitable interventions, and evaluate outcomes from tests of change [25]. Other valuable competencies related to QI are providing quality health care, striving for excellence in line with nurses’ professional values and ethical responsibilities, constantly improving nursing care, enhancing patient experiences and outcomes, and remaining effective and sustainable. However, pedagogical research focusing on QI education in nursing has been few and far between [12].

### Interactive QI examination

Participating students worked in four groups of three to five members. Assessments were conducted in an active learning classroom equipped with digital screens placed adjacent to each student group’s work area. The classroom had an open layout so that all student groups could hear and view each group’s PowerPoint presentations.

Before the examination, students were instructed to select and define an objective for their QI project—for instance, implementing a malnutrition screening policy or preventing pressure sores. The groups were also instructed to situate the project in a clinical context (e.g., nursing home or hospital ward), specify the type of care given, describe staff characteristics, and specify the number of patients.

During the examination, following the think–pair–share method [26], each student group was asked to present its QI project in order to receive feedback from other groups and lecturers, which enabled students to use higher-order thinking skills. To structure their presentations, the student groups used a template for a PowerPoint presentation (Supplementary File 2), and during the presentations, the lecturers used prompts to encourage students to elaborate on their thoughts and thereby progress in their learning.

Three rounds of presentations occurred during the examination:

1. During the first round, the groups presented the objectives of their projects according to SMART objectives (i.e., specific, measurable, applicable, realistic, and time-bound) [27];
2. During the second round, the groups presented their project diagrams, either as a flowchart or as a fishbone diagram (i.e., Ishikawa diagram); and
3. During the third round, the students presented a full project cycle following the structure in the plan–do–study–act model [28].

Based on students’ evaluations, we made three modifications between Cohorts 1 and 2 (Fig. 1):

- Individual response technology (Mentimeter) was used during the QI lecture to engage students and allow them to ask questions anonymously, following research indicating that such technology increases nursing students’ engagement and participation during lectures [29];
- A mandatory online quiz to qualify for the examination was implemented, because inadequate preparation by students before class is a major challenge for active learning pedagogy [8]; and
- The length of the interactive examination was extended by an hour—that is, to four hours total—to allow students to elaborate upon their projects more thoroughly.

For Cohort 3, we kept the modified course design used with Cohort 2 but conducted the lecture and examination online using Zoom’s videoconferencing platform. For that cohort, instead of a physical room, a virtual meeting room in Zoom was used for group presentations, and students worked in small groups to prepare for their presentations by using Zoom’s “Breakout rooms” function. Students were instructed that verbal and written (i.e., chat) modes of communication were comparable and that both modes of activity would be assessed equally during the examination. Lecturers also used the chat function to engage students in conversations by addressing their chat messages and asking them to elaborate.

### Evaluative questionnaire

To evaluate students’ experiences, we created a study-specific questionnaire, with questions seeking numerical and free-text responses. A panel consisting of a researcher in pedagogy, an associate professor in nursing, and a full professor in nursing validated the questionnaire. Six nursing students in their third semester and three other students majoring in other health care professions validated the questionnaire for face validity. After both validation processes, the questionnaire was revised accordingly. For an English translation of the questionnaire, please see Supplementary File 3.

For the campus-based examinations, Cohort 1 and 2, paper-based questionnaires were distributed at the end of the examination. Anonymity was secured because students did not include any identifiable information on the questionnaires, and they returned them in sealed envelopes. As researchers, we did not know if the participants filled out the questionnaires or not, and a few students submitted blank questionnaires in the envelopes (Fig. 1). For Cohort 3, an online version of the questionnaire was used, created in Microsoft Forms. Here, a link was distributed in the Zoom chat function at the end of the online examination which directed the students to the anonymous form. The form did not include any identifiable information and upon submitting the questionnaires, there was no way for us to track the forms to specific students.

### Variables

Questions about participants’ background requested their age (i.e., in yearly intervals) and gender (i.e., man, woman, or do not wish to disclose). One yes-or-no question asked whether students had attended or watched the QI lecture, whereas another asked whether they had participated in a previous QI project.

To evaluate the students’ overall experiences with the interactive examinations, we used one question—“Overall, how did you experience the examination?”—to be answered on a visual analogue scale, ranging from 0 (*very poor*) to 10 (*very good*). We also used three sets of statements addressing students’ engagement and preparedness, their learning achievements, and instructional aspects. A Likert scale, ranging from 1 (*strongly disagree*) to 5 (*strongly agree*), was used for all statements. Many of these statements have been previously used in another Swedish student evaluation study [30].

The first set of statements focused on engagement and preparedness: “I felt involved,” “I felt engaged,” “There were necessary prerequisites for me to be prepared,” “I felt prepared,” and “There was an open and permissive atmosphere.” The second set focused on learning achievements: “I am more aware of the value of QI in nursing,” “I have an increased understanding of QI in nursing,” and “I will apply QI in my role as a registered nurse.” Last, the third set focused on instructional aspects of the examination: “That we designed the quality improvement project ourselves,” “That we based the projects on personal experiences,” and “That we discussed and compared our projects as a group.”

We also obtained qualitative data from two free-text response boxes where students could write comments about the positive and negative aspects of the examination and how it could be improved.

### Data analysis

Our mixed-methods analysis focused on the quantitative findings, interpreted and illustrated with reference to qualitative results—that is, students’ descriptions of their experiences [19]. When selecting and presenting students’ descriptions, we observed three principles: that they represented the patterns in the data, corroborated our argument about the data, and were reasonably succinct [31].

We undertook descriptive statistical analysis with means of frequencies and percentages to identify patterns, similarities, and differences. Chi-squared tests were conducted to compare background variables between the cohorts. The 10-point visual analogue scale assessing students’ overall experiences was presumed to be continuous and means for that variable were used to compare the cohorts.

We used the Kruskal–Wallis H test, a nonparametric test appropriate for analyzing ordinal scales [32, 33], to compare the difference in ratings between cohorts. Although the test does not require data to be normally distributed, the homogeneity of variance between groups needs to be similar for assessment to be accurate. Thus, Levene’s test for the equality of variance based on item medians was conducted [34]. One item, “We worked in groups,” indicated significant unequal variance between the groups and was thus omitted from further analysis.

When differences in variance between cohorts were significant according to the Kruskal–Wallis H test, we performed post hoc tests (i.e., pairwise comparisons using Dunn’s test) to identify the groups for which the differences were significant. For pairwise comparisons, significance values were adjusted with Bonferroni correction for multiple tests [35]. Here, quartiles are used (i.e., Q1–Q3) to display item ratings for the three cohorts.

We used SPSS (v. 26, IBM) and JMP (v.15, SAS Institute) for all aspects of statistical analysis and considered *p*-values less than 0.05 to indicate statistical significance.

### Ethical considerations

This study was anonymous and did not collect sensitive personal information and therefore did not require formal approval from an ethics committee according to Sweden’s 2004 act concerning the Ethical Review of Research Involving Humans (SFS 2003:460). The department chair and the director of the nursing program reviewed and approved this study. We followed the guidelines outlined in the Declaration of Helsinki and students received information, both written and verbal, stating that completing the questionnaire was voluntary, anonymous, and that study results would be published. The students provided informed consent to participate by answering the questionnaire [32]. Ethical considerations also include understanding the power imbalance that exists between us as senior lectures and the students we are assessing. As this study was conducted following an examination, we made sure to emphasize that participation was voluntary, that we could not identify students’ responses, and that it thus would not influence our assessment of the students’ academic performances.

## Results

Of the 293 students who participated in the examination, 246 (84%) completed the questionnaire. Between the cohorts, response rates varied from 98% in Cohort 1 to 99% in Cohort 2 to 58% in Cohort 3 (the online cohort). Most participants were women, three-quarters were less than 31 years old, and a third had previous experience with participating in QI projects.

Cohort 1 consisted of proportionately more men than Cohorts 2 and 3 (*p* = 0.029). In Cohort 3, nearly all students (98%) had watched the online QI lecture, whereas fewer had in Cohorts 1 (81%) and 2 (89%) while attending the lecture on campus (*p* = 0.007). Otherwise, no significant differences in background variables surfaced between the cohorts (Table 1).

**Table 1:** Demographic and background information of participants (*n* = 246)

	Cohort 1 (*n* = 89)	Cohort 2 (n = 97)	Cohort 3 (n = 60)	
**Variables**	n (%)	n (%)	n (%)	**P-value**^a^
**Age in years**				0.780
20–25	44 (51)	44 (46)	27 (45)	
26–30	23 (27)	27 (28)	22 (37)	
31–35	10 (12)	11 (12)	5 (8)	
Over 35	8 (9)	12 (13)	4 (7)	
Do not wish to disclose	1 (1)	1 (1)	2 (3)	
**Gender**				0.029^*^
Woman	70 (82)	88 (94)	56 (93)	
Man	14 (16)	6 (6)	3 (7)	
Do not wish to disclose	2 (2)			
Attended/watched quality improvement lecture (Yes)	70 (81)	86 (89)	59 (98)	0.007**
Previous experience of quality improvement projects (Yes)	34 (40)	39 (41)	33 (55)	0.135

^a^ Chi squared test

* *p* < 0.05, ** *p* < 0.01

In this section, participants’ written comments are quoted along with information using the following key: C = cohort, ID = questionnaire ID, W = woman, M = man, and y = age in years.

### Students’ general experiences with the interactive examination

Students rated their overall experience with the interactive examination rather highly. On the 10-item scale, the sample as a whole (*n =* 246, all three cohorts) gave a mean score of 8.15.

Generally positive comments praised the examination’s educational and interactive aspects: “This way of learning is very rewarding. Discussing is helpful and educational because you get different perspectives since everyone has different knowledge and experiences” (C-2, ID: 2020, W, 30–35y). Other comments praised the examination’s focus on the process, not only the product: “[The experience was] Really great! I like that the examination focused on the process” (C-2, ID: 2066, M, 30–35y).

However, significant differences arose between the cohorts regarding their overall experiences with the examination (*p* < 0.001). On either side of Cohort 1’s overall mean score (8.12), Cohort 2 gave the highest score (8.56) and Cohort 3 the lowest (7.53). Significant differences also emerged between the cohorts regarding experiences with engagement (*p* = 0.023), preparedness (*p* = 0.000), and permissiveness (*p* = 0.002). However, students in Cohorts 1 and 2 gave significantly higher scores than students in Cohort 3 (Table 2).

**Table 2:** Nursing students’ general experiences of conducting the three interactive examinations in ALC

Item	Cohort (Q1,Q2,Q3)		H^1^	P-value
Overall, how did you experience the examination? (score: 1–10)			18.567	0.000**
	1 (7,8,9)	2		0.044*
	2 (8,9,9.5)	3		0.000**
	3 (7,8,8)	1		0.136
I felt involved during the examination			4.530	0.104
I felt engaged during the examination			7.585	0.023*
	1 (4,5,5)	2		1.000
	2 (4,5,5)	3		0.025*
	3 (4,4,5)	1		0.075
There were necessary prerequisites for me to be prepared			21.194	0.000**
	1 (4,5,5)	2		0.167
	2 (4,5,5)	3		0.000**
	3 (4,4,5)	1		0.014*
I felt prepared for the examination			15.086	0.001**
	1 (4,4,5)	2		1.000
	2 (4,4,5)	3		0.001**
	3 (3,4,4)	1		0.003**
There was an open and permissive atmosphere			11.986	0.002**
	1 (4,5,5)	2		0.005**
	2 (5,5,5)	3		0.022*
	3 (4,5,5)	1		1.000

1 = chi-square distribution

* = p < 0.05, ** = p < 0.01

Students’ written comments regarding preparation addressed the importance of being thoroughly prepared to pass the examination: “If we hadn’t prepared, then we wouldn’t have been able to complete our project” (C-1, ID: 1026, W, 20–25y). In Cohort 1, comments related to wanting more specific instructions about what and how to prepare: “I wish that we would’ve received more information about being better prepared” (C-1, ID: 1002, M, 26–30y) and “[I would have liked] A clearer picture of what to prepare” (C-1, ID: 1052, W, >35y). In Cohort 3, which had the lowest score for preparedness, comments reflected the need for more specific instructions due to the online format: “The syllabus could’ve been clearer, particularly now that it’s on Zoom, about how to best prepare for the examination” (C-3, ID: 3033, W, 20–25y).

In Cohorts 2 and 3, which required passing a digital quiz to qualify for the examination, many students commended the quiz for helping them in preparing for the examination: “The quiz was good because it forced you to prepare, which made the examination very educational and enjoyable” (C-2, ID: 2021, W, 20–25y). Other students appreciated the interrelatedness of the quiz, the learning activities, and the supporting documents: “The clear connection between the lecture, quiz, and syllabus made for a constructive way of learning” (C-2, ID: 2076, W, >35y).

Students often reflected on the process’s open, permissive climate: “This interactive way of taking exams contributed to an open, constructive learning climate” (C-2, ID: 2076, W, >35y). In Cohort 3, most comments about taking the examination online were positive: “I thought it went well on Zoom, with working in our groups in our rooms and then gathering and presenting. The number of students was suitable for Zoom” (C-3, ID: 3047, W, 26–30y). However, other students mentioned problematic technological aspects of online learning and personal preferences for campus-based education: “I experienced some technical issues; otherwise, it was OK” (Cohort 3, ID: 3020/206, W, 20–25y) and “It worked, but it would’ve been easier to do on campus” (C-3, 3009, M, >35y). Regarding involvement, others criticized the time constraints online: “It worked well on Zoom, but it would’ve been good to have more time with our groups in the breakout rooms” (C-3, ID: 3046, W, 20–25y).

### Students’ experiences with learning achievement

Concerning students’ experiences with their learning achievement, the only significant difference occurred between Cohorts 2 and 3 regarding their relative awareness of QI’s value in nursing after the examination (*p* = 0.028), as detailed in Table 3.

**Table 3:** Nursing students’ experiences of their learning achievements after conducting the three interactive examination designs in ALC

Item	Cohort (Q1,Q2,Q3)		H^1^	P-value
I am more aware of the value of QI in nursing			7.140	0.028*
	1 (4,5,5)	2		1.000
	2 (4,5,5)	3		0.025*
	3 (4,4,5)	1		0.161
I have an increased understanding of QI in nursing			4.208	0.122
I will apply QI in my role as registered nurse			3.529	0.171

1 = chi-square distribution

* = p < 0.05

While statistically significant differences emerged between Cohorts 2 and 3 on the topic of becoming more aware of QI’s value in nursing, no comments specifically reflected those differences. Instead, comments mostly addressed general aspects of having gained a better understanding of QI after the examination: “The examination was good and educational. I now feel more secure with participating in a quality improvement project ‘in real life’” (C-1, ID: 1007, W, 20–25y).

### Students’ experiences with the instructional aspects of the examination

Regarding the instructional aspects of the examination, all significant differences occurred between Cohorts 2 and 3, including about designing the projects (*p* = 0.022) and discussing and comparing them in groups (*p* = 0.010). Students in Cohort 2 generally scored the highest, whereas students in Cohort 3 scored the lowest (Table 4).

**Table 4:** Nursing students’ experiences of instructional aspects during the three interactive examination designs in ALC

Item	Cohort (Q1,Q2,Q3)		H^1^	P-value
That we designed the QI project ourselves			7.675	0.022*
	1 (4,5,5)	2		1.000
	2 (4,5,5)	3		0.019*
	3 (4,4,5)	1		0118
That we based the QI projects on personal experiences			5.608	0.058
That we discussed and compared our projects as a group			9.290	0.010*
	1 (4,4,5)	2		1.000
	2 (4,5,5)	3		0.008**
	3 (4,4,5)	1		0.065

1 = chi-square distribution

* = p < 0.05, ** = p < 0.01

Several students characterized the experiential approach as being educational: “It’s much more educational when you do something practical instead of just reading about it” (C-2, ID: 2032, W, 20–25y) and “It’s important to work with quality improvement in order to get a sense of how it really works; otherwise, it would be a bit vague or distant” (C-3, ID: 3022, W, 26–30y). Others described the educational process as somewhat trying: “People learn by trying, maybe doing it wrong, and relearning” (C-2, ID: 2016, W, 20–25y). The examination’s focus on process and interaction also justified some students’ praise for the online examination versus a written one: “[It was] Much more fun and educational [to share feedback based on personal experience] than take a written assignment” (C-1, ID: 1053, W, 20–25y).

Most comments about the examination’s instructional aspects addressed interacting within and between groups, and some emphasized the value of repeated discussions: “It was good to continuously discuss; otherwise, it would’ve been difficult to remember and learn” (C-2, ID: 2042, W, 26–30y) and “[It was] Very good to be continuously present. It made me concentrate more, so that I could process what was said during the discussions” (C-1, ID: 1031, W, 20–25y).

Comments from students in Cohort 3 underscored various aspects of interacting online, especially the benefit of the chat function: “The chat box worked very well. I could comment without interrupting the group that was presenting. Otherwise, I would have had to wait until the end of the presentation, when I might have forgotten my question or when it would have been strange and out of context” (C-3, ID: 3029, W, 26–30y). Another stated, “The chat box was good! It felt reassuring to know that I could participate even if I didn’t have the opportunity to join the verbal discussion” (C-3, ID: 3023, W, 30–35y).

Other students recommended improvements for the online interaction, including that lecturers should be more attentive and even moderate the chat: “It didn’t always feel like what people wrote in the chat got brought up for discussion. The discussion in the chat was also not always relevant to the subject” (C-3, ID: 3060, M, 30–35y). They also mentioned organizing the chat better: “Maybe consider organizing the questions in the chat, so that nothing gets lost” (C-3, ID: 3046, W, 20–25y) and “It could be difficult to get a word in edgeways if you’re not used to the format. It is difficult to know when you might enter the discussion” (C-3, ID: 3005, M, 26–30y).

## Discussion

In our study, we compared three interactive examination designs, all based on active learning pedagogy, in terms of nursing students’ engagement and preparedness, their learning achievement, and instructional aspects. The results indicate that the campus-based design with an active learning classroom was the design most appreciated by students. In particular, that design involved using a student response system during the QI lecture and an online qualifying quiz to sufficiently prepare students for the examination. A similar design conducted online, by contrast, received the overall lowest rating from students. Nevertheless, those students, especially ones with a fear of public speaking, largely praised the online design for allowing them to actively participate by means of written communication (i.e., digital chat).

Most of the significant differences, largely concerning students’ experiences with actively engaging during the examination, emerged between Cohorts 2 and 3. Differences between the cohorts regarding their experiences with learning achievement, however, were not as remarkable. Those findings suggest that the interactive campus-based examination particularly contributed to facilitating engagement, which upholds a fundamental aspect of active learning—that is, excitement—emphasized since the beginning of the concept’s development [3].

### Students’ preparation and fair assessment

Of all participants, students in Cohort 2 seemed to be the most prepared and have the best group-work dynamic, which their written comments corroborate. In that cohort and Cohort 3, using a qualifying online quiz seemed appropriate, which supports past findings that digital learning methods (e.g., quizzes and online simulations) are valuable methods of stimulating nursing students’ reflections and can promote self-correction [36]. In online learning environments, it is also important to consider both individual and social learning achievements [37]. By using individual quizzes along with group work during the examination, we could assess students’ individual and collaborative performance.

To make assessments fair, it was important to have two examiners compare and discuss students’ performance. For that purpose, having 20 students per group in four or five groups allowed intimate lecturer–student interaction and, in turn, discouraged students from engaging in a documented problematic behavior in group work: acting as freeloaders [14]. Compared with written examinations, the interactive assessments also allowed asking students follow-up questions that could reveal fundamental gaps in their understanding or else clarify and expand on their understanding, which facilitated making fair assessments of the depth or breadth of their knowledge. Thus, in line with previous studies, we believe that the design for Cohort 2, which integrated aspects of digital-based learning (e.g., digital quizzes) with face-to-face activities, cultivated an optimal learning style that sufficiently prepared students for the examination [36].

A particularly positive component—close lecturer–student interaction during the interactive examination—enabled teachers to discern whether and, if so, then how the examination captured aspects of students’ learning achievement that they wanted to assess. That direct feedback, together with students’ evaluations, indicated what needed to be revised in the course’s structure. As such, the examination also functioned as a pedagogical evaluation, one that would be difficult to undertake with a more summative examination (e.g., a written examination). This pertains to an advantage with formative assessments, as content saturation is a well-known concern in nursing education, and it is therefore important that nursing programs implement such educational methods with the potential to effectively examine and evaluate courses [15].

Despite significant differences in students’ experiences with the course layouts, the proportion of students per cohort who passed the examination—approximately 10% in each cohort—did not differ significantly, even though students in Cohorts 2 and 3 had to pass a digital qualifying quiz before attending the examination. Such differences may be explained by the fact that though the examination contents and objectives were identical, the conditions differed (e.g., Cohort 2 and 3 had one hour more than Cohort 1).

### Students’ involvement and active participation

Many students in Cohort 3 appreciated the digital chat function, which allowed them to actively participate without speaking aloud to the entire group. Research has shown that in online education, communication strategies should be built into the design and cannot assume the same conditions online as in face-to-face settings. Otherwise, when communication strategies are poorly defined or inappropriately applied, restrictions to synchronous and asynchronous learning occur [16]. Some of the students’ comments indicated that difference, either by applauding the function that allowed written feedback and comments or criticizing such parallel communication as distracting.

Research has also revealed that exposure to virtual environments can boost students’ confidence and enable them to face audiences of any size [38], which especially benefits students with a fear of public speaking. Such confidence is crucial for nursing students, who generally need to practice playing active roles for their profession and engage in QI work as an important skill for enhancing the quality of care and patients’ safety [39]. Nurses should also be able to safeguard the interests of vulnerable patients during care planning processes and in meeting with interprofessional teams [40, 41]. Added to that, registered nurses, who are typically expected to be clinical leaders for nursing aides and assistants, need to be confident and professional in their interpersonal communication, which will most likely occur in face-to-face interactions [42]. In that light, while nonverbal communication enabled certain students to achieve the learning objective of active participation during the examination, the chat function allowed students, especially shy ones, to engage verbally since lecturers could address their written comments and ask them to elaborate.

Overall, the students’ evaluations indicate that the examination’s active focus on the process, commonly expressed as “doing” in the comments, contributed to their learning. It is precisely when students engage in activities—for example, design a project and present it—that they gain opportunities for higher-order thinking and that deep learning and retention are most likely to occur [43]. We also believe that such doing was reflected in both the product (i.e., the QI project) and the process of working in groups, the latter of which students characterized as motivating, arguably because we instructed students to center their projects in well-defined clinical contexts. Group work is often most motivating when perceived by students to have a meaningful, real-world context and/or implications [14]. In that light, students should base their QI projects on problems experienced during clinical placements or clinical work. As a result, aside from content, the processes underlying the assessment of group work skills (e.g., collaboration and negotiation) and other employable skills can be perceived as authentic [44].

### Strengths and limitations

Among our study’s multiple strengths and limitations, a chief strength was the high response rates in Cohorts 1 and 2, most likely because those students used a paper-based questionnaire. As a result, we had groups of participants that were representative in terms their age and gender distribution. In Cohort 3, in which students completed the examination online, the response rate was significantly lower, which confirms that online questionnaires tend to have lower response rates than their paper-based versions [45]. Additionally, our students are exposed to many questionnaires during their education through their online teaching platform (e.g., course evaluation forms), and privately because of the emerging internet-based services that collect data. Research also indicates that the quick shift to online education warranted by the COVID-19 pandemic in general can induce a fatigue among students [46]. This might have influenced the students’ engagement to complete an online form after the examination. It might also be that the teacher-student relationship were not as intimate during online education which might make students less inclined to complete evaluative questionnaires. The results from Cohort 3 are thus prone to selection bias, which limits the generalizability and comparability of this cohort.

Another limitation was that the evaluative questionnaire was not psychometrically tested beyond face validity. However, we did use the same questionnaire with the same questions and layout in all cohorts, only that the mode of delivery for Cohort 3 was online. Such conditions thus likely enabled a reliable comparison of the cohorts.

At the same time, following the GRAMMS guidelines was a strength of our study. It allowed us to not only report research adequately so that all readers can critically appraise our study but also aid systematic reviewers in identifying key methodological issues in the literature.

Last, a potential limitation with mixed-methods designs is the possibility of contradictory findings—for example, when different data do not fully support each other [19]. However, in our study, no contradictions in the data were apparent; in fact, the two data sources (i.e., numeric, and free-text responses) complemented each other well. For example, regarding experiences with preparedness, students in Cohort 3 had the lowest numerical scores despite taking the qualifying digital quiz, unlike the students in Cohort 1. The written comments highlighted that many experiences with being poorly prepared related to the digital mode of delivery, not to the content, as was the focus of the digital quiz. Thus, the written comments supported a nuanced understanding of the differences and understanding of the numerical ratings.

### Implications and future research

Our results generally show that interactive examinations are feasible and appreciated by nursing students in their final year of study. Above all, to conduct such examinations successfully, educators need to focus on students’ preparation. To that end, they should consider using the strategies described in this article, which prioritized incorporating different digital technologies as resources and administering digital quizzes to complement students’ group work.

Several implications can be drawn from the experiences captured by the study. First, using individual response technology during lectures is one way to engage students [29]. We used Mentimeter but several brands and products (e.g., clickers) are also available [47]. Research has shown that such approaches allow students to provide input without fear of public embarrassment or having to worry about being sidelined by more outspoken students [47, 48]. That type of active participation and interaction is particularly important in preparing for interactive examinations, which often expect students to actively participate in order to pass.

Second, the online quiz contained questions that clearly reflected the course objectives, all aimed to guide students in navigating the learning objectives. In response, students described the help as having supported their preparation, and other research has indeed shown that students can consult quizzes to direct their independent learning [49]. Beyond that, because the quiz consisted mostly of multiple-choice questions and was self-graded, it was time-efficient for us as lecturers.

Third, educators have a range of videoconferencing platforms to choose from, including Zoom, Skype, and Microsoft Teams. When using such platforms, it is important to consider so-called “netiquette” by, for example, establishing clear guidelines for behavior and providing information about technical requirements. In our study, the links to meetings on Zoom were accessible only via the student learning platform, which is accessible to enrolled students only. We also enabled the “Waiting room” function to admit students one at a time, which allowed us to confirm their identities before they entered the meeting. On many videoconferencing platforms, it is also possible to create public links but restrict access with passwords that can be distributed internally. Those security aspects are important given research revealing students’ concerns with the integrity of online examinations [50].

Fourth, significantly more students in Cohort 3 had watched the online QI lecture than students attending the campus-based lectures in Cohorts 2 and 3. That result suggests that implementing a blended learning model (e.g., using both campus- and online-based educational activities in courses) might facilitate student attendance and engagement, and in turn, better support their preparation before examinations.

In light of those implications, future studies should evaluate educators’ experiences with interactive examinations conducted online. Research has shown that educators experience several challenges in transitioning from in-class lessons to online-based ones, including a lack of technological support and the need for professional development [51]. A recent review has additionally indicated that few studies concerning digital technologies in higher education have involved evaluating interpersonal communication and collaborative learning from the perspective of students [52]. Thus, as we intended in our study, research in the future should also consider those experiential interpersonal aspects of online education.

## Conclusion

Students’ experiences with interactive examinations, can be enhanced when the assessments have campus-based designs, focus on students’ preparation, and provide students with sufficient time to complete the examination. To continue improving the quality of teaching and enhance learning achievement by using new technologies with students, it is essential for teachers to identify aspects of their teaching practices that can be improved. To those ends, the interactive methods of assessment presented in our study possess several advantages.

## Supplementary Information

**Additional file 1.** Good Reporting of a Mixed Methods Study (GRAMMS)^1^ checklist.

**Additional file 2.** An illustration of the power point presentation template that the nursing student groups used during the interactive examinations.

**Additional file 3.** English translation of the study-specific questionnaire questions.

## Data Availability

The datasets used and analyzed during the current study are available from the corresponding author on reasonable request.

## Abbreviations

ALC: Active Learning Classroom
GRAMMS: Good Reporting of a Mixed Methods Study
