# Supplementary material for: A comparison of three interactive examination designs in active learning classrooms for nursing students
Source: BMC Nurs. 2021 Apr 9;20:59. doi: 10.1186/s12912-021-00575-6 (PMC8033549; doi:10.1186/s12912-021-00575-6)
Supplement: Supplementary file 1 — Additional file 1. Good Reporting of a Mixed Methods Study (GRAMMS)1 checklist. [file 12912_2021_575_MOESM1_ESM.docx]

**Manuscript title**

A COMPARISION OF THREE INTERACTIVE EXAMINATION DESIGNS IN ACTIVE LEARNING CLASSROOMS FOR NURSING STUDENTS

**Running title**

A COMPARISON OF THREE INTERACTIVE EXAMINATION DESIGNS

**Authors**

Ahlstrom, Linda^1,2^, Holmberg, Christopher^1,3^

1. Institute of Health and Care Sciences, Section of Learning and Leadership for Health Care Professionals, University of Gothenburg, Arvid Wallgrens Backe, Box 457, 405 30. University of Gothenburg, Sweden.
2. Department of Orthopedics, Sahlgrenska University Hospital, Gothenburg, Sweden
3. Department of Psychotic Disorders, Sahlgrenska University Hospital, Gothenburg, Sweden

*Credentials*

Linda Ahlstrom (LA), RN, PhD, Assistant Professor

Christopher Holmberg (CH), RN, PhD, Assistant Professor

**Corresponding author**

Dr. Christopher Holmberg, [christopher.holmberg@gu.se](mailto:christopher.holmberg@gu.se)

Tel: +46 (0) [766-18 18 52](tel:+46766181852)

Institute of Health and Care Sciences, Section of Learning and Leadership for Health Care Professionals, University of Gothenburg, Arvid Wallgrens Backe, Box 457, 405 30. University of Gothenburg, Sweden.

**Good Reporting of a Mixed Methods Study (GRAMMS)^1^ checklist**

| **Guideline** | **Section in manuscript** |
| --- | --- |
| Describe the justification for using a mixed-method approach to the research question | Design. |
| Describe the design in terms of the purpose, priority and sequence of methods | Data analysis. |
| Describe each method in terms of sampling, data collection and analysis | Design, Data analysis. |
| Describe where integration has occurred, how it has occurred and who has participated in it | Design, Interactive QI examination, Evaluative questionnaire, Variables, Data analysis. |
| Describe any limitation of one method associated with the presence of the other method | Strengths and limitations |
| Describe any insights gained from mixing or integrating methods | Strengths and limitations |

^1^ O'Cathain A, Murphy E, Nicholl J. The quality of mixed methods studies in health services

research. J Health Serv Res Policy. 2008;13: 92-98.
