# Supplementary material for: A comparison of three interactive examination designs in active learning classrooms for nursing students
Source: BMC Nurs. 2021 Apr 9;20:59. doi: 10.1186/s12912-021-00575-6 (PMC8033549; doi:10.1186/s12912-021-00575-6)
Supplement: Supplementary file 2 — Additional file 2. An illustration of the power point presentation template that the nursing student groups used during the interactive examinations. [file 12912_2021_575_MOESM2_ESM.docx]

**Manuscript title**

A comparison of three interactive examination designs in active learning classrooms for nursing students

**Running title**

A comparison of three interactive examination designs

**Authors**

Ahlstrom, Linda^1,2^, Holmberg, Christopher^1,3^

1. Institute of Health and Care Sciences, Section of Learning and Leadership for Health Care Professionals, University of Gothenburg, Arvid Wallgrens Backe, Box 457, 405 30. University of Gothenburg, Sweden.
2. Department of Orthopedics, Sahlgrenska University Hospital, Gothenburg, Sweden
3. Department of Psychotic Disorders, Sahlgrenska University Hospital, Gothenburg, Sweden

**Corresponding author**

Dr. Christopher Holmberg, [christopher.holmberg@gu.se](mailto:christopher.holmberg@gu.se)

Tel: +46 (0) [766-18 18 52](tel:+46766181852)

Institute of Health and Care Sciences, Section of Learning and Leadership for Health Care Professionals, University of Gothenburg, Arvid Wallgrens Backe, Box 457, 405 30. University of Gothenburg, Sweden.

**Supplementary file 2.** An illustration of the power point presentation template that the nursing student groups used during the interactive examinations.
