# Supplementary material for: A comparison of three interactive examination designs in active learning classrooms for nursing students
Source: BMC Nurs. 2021 Apr 9;20:59. doi: 10.1186/s12912-021-00575-6 (PMC8033549; doi:10.1186/s12912-021-00575-6)
Supplement: Supplementary file 3 — Additional file 3. English translation of the study-specific questionnaire questions. [file 12912_2021_575_MOESM3_ESM.docx]

**Manuscript title**

A comparison of three interactive examination designs in active learning classrooms for nursing students

**Running title**

A comparison of three interactive examination designs

**Authors**

Ahlstrom, Linda^1,2^, Holmberg, Christopher^1,3^

1. Institute of Health and Care Sciences, Section of Learning and Leadership for Health Care Professionals, University of Gothenburg, Arvid Wallgrens Backe, Box 457, 405 30. University of Gothenburg, Sweden.
2. Department of Orthopedics, Sahlgrenska University Hospital, Gothenburg, Sweden
3. Department of Psychotic Disorders, Sahlgrenska University Hospital, Gothenburg, Sweden

**Corresponding author**

Dr. Christopher Holmberg, [christopher.holmberg@gu.se](mailto:christopher.holmberg@gu.se)

Tel: +46 (0) [766-18 18 52](tel:+46766181852)

Institute of Health and Care Sciences, Section of Learning and Leadership for Health Care Professionals, University of Gothenburg, Arvid Wallgrens Backe, Box 457, 405 30. University of Gothenburg, Sweden.

**Supplementary file 3.** English translation of the study-specific questionnaire questions.

**Your general experiences of the examination:**

|  | Strongly disagree |  | | | Strongly agree |
| --- | --- | --- | --- | --- | --- |
|  | 1 | 2 | 3 | 4 | 5 |
| I felt involved during the examination |  |  |  |  |  |
| I felt engaged during the examination |  |  |  |  |  |
| There were necessary prerequisites for me to be prepared |  |  |  |  |  |
| I felt prepared for the examination |  |  |  |  |  |
| There was an open and permissive atmosphere |  |  |  |  |  |

**Your experiences of learning achievements:**

|  | Strongly disagree |  | | | Strongly agree |
| --- | --- | --- | --- | --- | --- |
|  | 1 | 2 | 3 | 4 | 5 |
| I am more aware of the value of quality improvement (QI) in nursing |  |  |  |  |  |
| I have an increased understanding of QI in nursing |  |  |  |  |  |
| I will apply QI in my role as registered nurse |  |  |  |  |  |

**Your experiences of valuable instructional aspects:**

|  | Strongly disagree |  | | | Strongly agree |
| --- | --- | --- | --- | --- | --- |
|  | 1 | 2 | 3 | 4 | 5 |
| That we designed the QI project ourselves |  |  |  |  |  |
| That we based the projects on personal experiences |  |  |  |  |  |
| That we worked in groups |  |  |  |  |  |
| That we discussed and compared our projects as a group |  |  |  |  |  |

**What was the main positive and negative aspects of the examination?**

|  |
| --- |

**How can this examination be improved?**

|  |
| --- |

**Overall, how did you experience the examination? (circle a number from 0-10):**

**0 1 2 3 4 5 6 7 8 9 10**

**Very poor Very good**

**Did you attend the lecture about quality improvement?**

|  |  |
| --- | --- |
| **Yes** | **No** |

**Your age:**

|  |  |  |  |  |  |
| --- | --- | --- | --- | --- | --- |
| **Below 20 years old** | **20-25 years** | **26-30 years** | **31-35 years** | **Over 35 years** | **Do not wish to disclose** |

**Your gender:**

|  |  |  |
| --- | --- | --- |
| **Woman** | **Man** | **Do not wish to disclose** |

**Do you have previous experience of participating in quality improvement projects?**

|  |  |
| --- | --- |
| **Yes** | **No** |
